# Current Status for Controlling the Overlooked Caprine Fasciolosis

**DOI:** 10.3390/ani11061819

**Published:** 2021-06-18

**Authors:** Gemma Zerna, Terry W. Spithill, Travis Beddoe

**Affiliations:** Department of Animal, Plant and Soil Sciences and Centre for AgriBioscience, La Trobe University, Bundoora, Victoria 3083, Australia; gmzerna@students.latrobe.edu.au (G.Z.); T.Spithill@latrobe.edu.au (T.W.S.)

**Keywords:** *Fasciola hepatica*, *Fasciola gigantica*, goats, prevalence, susceptibility, vaccines, anthelmintics, control

## Abstract

**Simple Summary:**

Retaining sustainable agricultural systems is essential to feed the expanding population. Helminth parasite infections impact livestock production values and yields, including infections in goats, which are often overlooked. There is a requirement to collate caprine-specific knowledge surrounding fasciolosis, caused by *Fasciola hepatica* and *Fasciola gigantica* (liver flukes). Current liver fluke control methods include drug application and pasture management. This review aims to outline goat-specific fasciolosis vaccine development and the potential for alternative control methods.

**Abstract:**

The disease fasciolosis is caused by the liver flukes *Fasciola hepatica* and *F. gigantica*, which infect a wide range of mammals and production livestock, including goats. These flatworm parasites are globally distributed and predicted to cost the livestock industry a now conservative USD 3 billion per year in treatment and lowered on-farm productivity. Infection poses a risk to animal welfare and results in lowered fertility rates and reduced production yields of meat, milk and wool. This zoonotic disease is estimated to infect over 600 million animals and up to 2.4 million humans. Current and future control is threatened with the global emergence of flukes resistant to anthelmintics. Drug resistance calls for immediate on-farm parasite management to ensure treatments are effective and re-infection rates are kept low, while a sustainable long-term control method, such as a vaccine, is being developed. Despite the recent expansion of the goat industry, particularly in developing countries, there are limited studies on goat-focused vaccine control studies and the effectiveness of drug treatments. There is a requirement to collate caprine-specific fasciolosis knowledge. This review will present the current status of liver fluke caprine infections and potential control methods for application in goat farming.

## 1. Introduction

Parasite control is an essential strategy for retaining sustainable agriculture. Exacerbating the requirement for optimal production systems is the need to feed the expanding global population and the urbanisation that is encroaching on agricultural land. This will increase the pressure on agricultural systems to produce higher yields with potentially less space. There is a current health-conscious consumer mindset that has led to increased consumption and popularity of goat meat because of its nutritional benefits over other types of red meat. Chevon is lower in total fat, saturated fatty acids, and cholesterol content, making it a healthy protein source. Goat production has expanded substantially, in developing countries in particular, where there is a reliance on small local farming systems that can be significantly impacted when their herd is affected by parasites.

Helminth parasite infections can significantly reduce multiple aspects of animal production, including animal health, animal welfare, production yields and quality, along with a farmer’s net profit. The liver flukes, *Fasciola hepatica* and *Fasciola gigantica*, are highly successful flatworm parasites that have a tremendous ability to infect and thrive within a vast range of mammals, including humans. The disease caused by these flukes, termed fasciolosis, damages the liver and bile ducts of livestock, resulting in significant production losses and animal welfare issues. Controlling the disease is becoming paramount with the increasing prevalence of anthelmintic-resistant parasite populations [1,2]. Controlling infection effectively can amount to limiting the intermediate host snail environment, anthelmintic utilisation, potentially using natural fungal and plant extracts and, ideally in the future, vaccination.

## 2. Severity of Fasciolosis and the Infection Cycle

Fasciolosis, caused by *Fasciola* spp., is found on all five continents of the globe and is now recognised as a human neglected tropical disease by the World Health Organisation (WHO) [3]. Human infection is predominantly isolated to developing countries (Africa, South America, North and South Asia, China and Korea) where there is a higher chance of exposure to the infective fluke stage during food preparation. This led to around 17 million people being infected and another 180 million people at risk of acquiring liver fluke infections [4,5]. Fasciolosis in ruminants is widespread and causes significant impacts to the livestock industry, with a now conservative USD 3.2 billion per annum lost globally from reduced production yields and associated treatment costs [6,7]. Recent findings have determined that fasciolosis costs USD 15 million in Iran alone from cattle, sheep and goat infections [8]; furthermore, infected cattle in Brazil cost USD 35 per head due to the significant 5.8% reduction in carcass weight [9].

Traditionally, *F. hepatica* is prevalent in more temperate regions and the larger *F. gigantica* in the tropical areas. Sub-tropical regions can foster both species, and overlapping populations have subsequently led to hybrids appearing in Korea [10], China [11], Thailand [12], Vietnam [13], Iran [14], Japan [15], Pakistan [16] and Egypt [17]. Adult *F. hepatica* and *F. gigantica* are morphologically similar; both are leaf-like hermaphroditic flatworms at 20–50 mm for *F. hepatica* and 24–76 mm for *F. gigantica*, with less pronounced shoulders [18]. It is not easy to distinguish between *Fasciola* spp. eggs as they are both large operculated ovals around 130–150 μm in length with a yellowish-brown colour [18]. *Fasciola* spp. parasitise a wide range of domesticated livestock and wildlife, including but not limited to sheep, goats, cattle, buffalo, pigs, horses, camelids, donkeys, deer, rabbits, hares, antelope, giraffes, and zebras. Infections of the definitive host can result in reduced fertility, abortions, slowed growth rates, and reduced production of milk, meat and wool, along with contaminated livers and withered carcasses [19]. Infection infrequently causes mortality, but deaths have been observed in both sheep and goats [20,21].

Liver flukes have a complex life cycle, requiring an intermediate snail host for larval development before reaching their definitive host, where the adults reside and can thrive for many years [4,22]. Parasitic eggs laid by the adults within the animal host bile ducts are released into the surrounding environment through defecation. These eggs lay dormant until the optimal temperature and humidity conditions occur, then they embryonate and release motile miracidium through the then opened operculum. The ciliated miracidium has a limited life span with only 12 h to swim and locate a suitable mollusc intermediate host [18]. Fluke transmitting snails are of the genus *Lymnaea*, commonly *Galba* spp. for *F. hepatica* and *Radix* spp. and *Lymnaea* spp. for *F. gigantica* [23]. The continuation of the parasite life cycle is therefore dependent on adequate snail populations, which are influenced by environmental factors such as temperature, light, vegetation, water depth, current soil composition and competent snail populations [19]. Within the amphibious snail body, the parasite reproduces asexually, developing from a miracidium into a sporocyst containing daughter rediae and finally releasing cercariae. There is high parasitic replication within the snail; a snail infected with a single miracidium can shed hundreds of cercariae in waves over a period of several days to multiple months [24]. The free-living cercariae swim and settle upon aquatic vegetation, where they subsequently lose their tail and encyst as a metacercaria.

The definitive host ingests the infective metacercaria attached to the vegetation [22]. Within the small intestines, the metacercarial cyst is activated, and newly excysted juveniles (NEJs) are released. The NEJs then cross the intestinal wall into the peritoneal cavity and can spend several days migrating towards the liver [25]. Immature flukes transverse the liver parenchyma feeding on blood and tissue, causing extensive haemorrhaging and liver fibrosis. After approximately 6 weeks within the liver, the flukes migrate to the bile ducts and sexually mature into adults that produce up to 20,000 eggs per day [22,26]. The eggs are then carried by the bile to the duodenum and leave the body via the animal’s faeces, thus completing the life cycle.

## 3. Prevalence of Caprine Fasciolosis

The predominant focus of fasciolosis in ruminants has been on cattle and sheep infections due to their high economic importance and perceived higher profitability. However, on a global scale, total goat populations have had the most significant increase in recent years (2006–2016), with population growths of 19.3% compared to the almost 7% growth of both the cattle and sheep population [27]. Asia and Africa are the largest producers of goat meat, contributing to 95% of the global chevon production [28]. Prevalence estimates of fasciolosis in these regions range from 0.28–68.4% in Africa and 0.0–47% in Asia [6]. In the Americas, the highest level of fasciolosis was seen in goats (24.5–100%) compared to cattle, sheep and buffalo, with notable prevalence in Argentina and Mexico [6,29,30]. An exciting investigation into mixed parasite infections in Argentina revealed that 46% of Creole goats had mixed infections, and while *F. hepatica* was previously overlooked in Argentina; they showed a 33% prevalence in 659 Creole goats assessed (Table 1) [29]. From Europe, one study shows 3.8–15.9% prevalence in Greek indigenous goat breeds, while another study predicts an impact of EUR 86 million in losses due to *F. hepatica* infections in European dairy goats [31,32].

**Table 1:** Global prevalence of fasciolosis in goats and any associated economic values.

Country	Fasciola Species	Goat Breed	Study Period	Sample Number and Type	Prevalence	Comments	Ref.
Argentina	*F. hepatica*	Creole	2006–2011	659 faecal	33%	46% of goats had mixed parasitic infections	[29]
Mexico	*F. hepatica*	-	-	1,199 faecal	24.5–43%	43% from indirect ELISA 24.5% of faecal egg counts	[30]
Greece	*F. hepatica*	*Capra Prisca* and Skopelos	2006–2007	234 faecal 372 serum	3.8–15.9%	3.8% of faecal from coproantigen 15.9% seropositivity	[31]
Africa							
Chad	*F. gigantica*	-	2011	616 livers	12%	Of infected 80% had <10 parasites and 2% with >100 parasites	[33]
Egypt	*F. hepatica*	-	2019	1630 livers	3.5%	Assiut and Sohag Governorates, Upper Egypt	[34]
Ethiopia	*Fasciola* spp.	-	2010–2011	384 livers	13.6%	Debre Zeit town	[35]
Kenya	*F. gigantica*	-	1989–2004	17,743 livers	6.6%	Semi-arid coastal area of Taveta Cost USD 12,600 from contaminated livers	[36]
Tunisia	*F. hepatica*	-	2004–2005	19 sera	68.4%	Gafsa oases, Southwest Tunisia Goat prevalence was a lot higher than sheep and cattle	[37]
Algeria	*F. hepatica*	-	2008–2009	6115 livers	0–2.5%	2.5% in El Tarf, North Algeria from 5,764 livers 0% Ouargla, South Algeria 351 livers EUR 60,000 lost in El Tarf from fasciolosis (cattle, sheep and goats) condemned livers	[38]
Nigeria	Mostly *F. gigantica*	-	1993–2019	376,507 reports	1.28%	USD 27 million per year lost from mortalities, liver condemnation and body weight loss	[39]
	*F. gigantica*	-	2004–2009	9,617 livers	0.28%	Higher infection in rainy/dry season	[40]
Asia							
China	*Fasciola spp.*	-	2013–2014	200 faecal	3.5–37%	Hubei province Lowest prevalence (3.5%) during May 2014 Highest prevalence (37%) during May 2013	[41]
	*F. gigantica* and *F. hepatica*	-	2011	104 faecal	26%	Yunnan province Investigation after human *F. gigantica* infection	[42]
India	*F. gigantica*	-	2001–2004	12,741 faecal 812 livers	2.35–4.68%	North India 2.35% of faecal samples 4.68% of livers	[43]
	*F. gigantica*	-	2001–2004	3,956 faecal	2.02%	Uttar Pradesh area	[44]
	*Fasciola* spp.	-	-	300 faecal 90 GI tracts	10.97–12.87%	Patna, Bihar 12.87% of faecal samples 10.97% from gastrointestinal (GI) tract examination	[45]
Nepal	*Fasciola* spp.	-	2014	100 faecal	47%	Mahottari district had high prevalence	[46]
Bangladesh	*F. gigantica*	Black Bengal	2007–2008	325 livers	21.54%	Sylhet district	[47]
	*F. gigantica*	Jamnapari and Black Bengal	2016–2017	102 livers	10.84–15.79%	Rajshahi metropolitan area 15.79% Jamnapari (of 19) 10.84% Black Bengal (of 83)	[48]
	*F. gigantica*	Black Bengal	2007–2008	318 livers	20.75%	Sylhet district USD 115 per 1000 goat livers	[49]
	*F. gigantica*	Black Bengal	2014–2015	26,443 livers	3.82%	High prevalence in Kushtia, Jhinaidah and Rajbari USD 2,375 lost from liver condemnation	[50]
Turkey	*F. hepatica*	Hair goats	2018	580 livers	14.14%	Siirt province	[51]
Iran	*Fasciola* spp.	-	2015–2019	-	1.56%	Loss of USD 13.8 million from condemned sheep and goat livers 910,282 positive goats and sheep livers	[8]
	*F. hepatica*	-	1999–2008	400,695 livers	2.79%	Khuzestan, Southwest Iran	[52]
	*Fasciola* spp.	-	2012–2013	151,924 livers	2.76%	Kashan, Center of Iran USD 30,240 annually from contaminated livers	[53]
Pakistan	*Fasciola* spp.	-	2004–2005	252 faecal	0%	Rawalpindi and Islamabad regions	[54]
	*F. hepatica*	-	2007	300 faecal	10%	Lahore area	[55]
	*F. hepatica*	Beetal goats	2010	200 faecal 200 bile	2–4%	Punjab districts 2% of faecal and 4% of bile samples via microscopy	[56]

Goats are a favoured ruminant in Africa for their ability to survive extreme environments, withstand water deprivation and browse on leaves and bushes in arid African conditions [57]. Africa has a high population of Boer goats for meat production, and in 2016 there was an estimated 387 million goats in Africa, with a 37.6% increase in numbers from the recorded number in 2006 [27,28]. In Chad in Central Africa, goats had a similar prevalence and infection intensities as sheep, with 12% of 616 goats positive for infection after post-mortem examination with the majority (80%) having light infections, less than 10 parasites, but 2% with greater than 100 parasites [33]. There was a survey of fluke prevalence in Upper Egypt determining that of 1630 goat livers, 3.5% had current fluke infections [34]. In Ethiopia, the prevalence of caprine fasciolosis was lower than bovine and ovine disease, with 13.6%, 28.6% and 20.8%, respectively, of 384 for each animal assessed after slaughter [35]. Although not determined in the aforementioned study, there is a possibility of mixed *F. hepatica* and *F. gigantica* infections in the Ethiopian goats as mixed infections have been observed in sheep infections in Ethiopia around the same time period [58]. In a 15-year study from 1989–2004 in slaughtered animals in Kenya, there were over 1000 cases of *F. gigantica* infections sitting at 6.6% of the 17,743 goats killed, costing USD 12,600 from the loss of contaminated livers [36]. A survey on livestock fasciolosis in Tunisia was conducted after the occurrence of human infection; they found the highest prevalence in goats, 68.4%, compared to sheep (35%), cattle (14.3%) and humans (6.6%), and suggested that the high number of goats infected could have contributed to the human cases [37]. The neighbouring country, Algeria, had 0–2.5% prevalence and an estimated EUR 60,000 loss from cattle, sheep and goat fasciolosis in the northern region [38]. It is difficult to truly equate the economic impact and prevalence of fasciolosis infection in goats as the reports often combine data from goat and sheep infections or combine the costs of several parasite infections in goats. One of the few studies depicting solely caprine fasciolosis determined that USD 27 million per year is lost in Nigeria from mortalities, liver condemnation and the loss of body mass from the chronic disease [39].

Fasciolosis is widespread in Asia, but there are a surprisingly low number of reported caprine infections, despite the most extensive global populations of goats being in India and China [6,28]. There are limited surveys of fasciolosis in China, but the prevalence has been illustrated in sheep (28.5%), buffaloes (87.35%), Yaks (28.7%) and humans in different regions [42,59,60,61]. Goat infections have been noted in the Hubei and Yunnan province of China, with a prevalence of 3.5–37% and 26%, respectively [41,42]. This is a surprisingly low number of reported cases considering China has the most significant total percentage of goats (14.85%) with over 148 million head [28]. India has the second-highest goat population, 13.35% of the global population [28], and also a low number of reported cases of caprine fasciolosis, only isolated to north India. From abattoir studies, 4.68% of goats showed infections and 2.02–2.35% of goats were positive for *Fasciola* eggs during 2001–2004 in India, with an additional 12.87% goats containing eggs in their faeces in Patna and Uttar Pradesh in subsequent years [43,44,45].

Livestock plays a vital role in the economy in Bangladesh, and parasitism significantly impacts production as *F. gigantica* infects 3.82–21.54% of Black Bengal goats and 15.79% of Jamnapari goats, costing USD 2,375 or USD 115/1000 goat livers, depending on the district, which are considerable costs when taking into account the 56 million goats in Bangladesh in 2016 [27,47,48,49,50]. Within the Siirt region of Turkey, there was a 14% prevalence of caprine fasciolosis in 2018 [51]. The extensive study on the over 3 billion livestock slaughtered from 1999–2008 in Southwest Iran discovered that 11,181 of 400,695 (2.79%) goats were infected with *F. hepatica* [52]. Iran had increased condemned livers during winter, whereas cattle showed the highest infection levels during summer; this contrasting infection intensity in cattle and goats was also found in India [43,52]. A subsequent study in Central Iran from 2012 to 2013 showed a similar prevalence of 2.76% in goats, with 4,200 infected goat livers from 151,924 animals costing USD 30,240 annually from contaminated goat livers [53]. The prevalence of fasciolosis in goats within Pakistan was interestingly 0% in 2004–2005, and subsequently 10% in 2007–2008, then 2–4% in 2010, all from different regions of Pakistan notably using varying detection methods [54,55,56].

Fasciolosis is a global disease that infects a large number of goats, and it is likely that the total number of infected goats is only partially reflected here, considering the lack of data obtained from areas with high goat populations. More extensive epidemiological investigations that do not neglect goats are needed to truly gauge the severity of caprine fasciolosis.

## 4. Susceptibility of Goats to Fasciolosis 

Goats are highly susceptible to fluke infections, with no apparent evidence of establishing acquired resistance to *F. hepatica* infections. However, goats do have potential capabilities to elicit protective immunity towards *F. gigantica* infections [62,63]. The current information indicates that caprine infections and susceptibility align with the high susceptibility of ovine infections rather than the partial resistance observed in bovine infections. The high susceptibility of goats towards *F. hepatica* infection is inferred from the lack of fluke rejection during primary exposure, along with a lack of evidence for acquired immunity upon secondary infection with rapid advancement to patency of parasites on re-exposure [62,64].

It is apparent that both host and parasite factors are intertwined in developing parasite resistance. Cattle display some resistance to *F. hepatica* as they can clear a primary infection within 7 months and are capable of diminishing parasite burdens upon re-exposure, potentially through immunological mechanisms [25,65,66]. The lowered severity of infection levels in cattle upon secondary *F. hepatica* infection is dependent on the duration of the primary exposure, where a short primary exposure (7 weeks) is unable to elicit protection, but longer (>12 weeks) exposure can reduce parasite levels [25,66]. Further exploration into acquired immunity in cattle showed that exposure to the juvenile parasite from either one or three 5-day drug-abbreviated infections can confer protection, as can inoculation with irradiated metacercariae [7,67,68]. Generally, sheep are susceptible to re-infection as there is a lack of evidence for the development of acquired immunity [65,68]. While there is no clear evidence for acquired immunity, certain sheep breeds can display lowered disease severity with reduced liver damage, parasite size and egg output after a primary fluke exposure [68,69]. Susceptibility to *F. gigantica* infection varies across the different sheep breeds; fluke infection of the Dorper and Red Masai breeds resulted in significantly higher faecal egg counts in the Dorper breed along with higher parasite numbers, although not significantly [70]. Additionally, after *F. hepatica* infection in five sheep breeds, the Barbados Blackbelly was determined as the most susceptible after both primary and re-exposure challenges as it had the highest fluke burdens and egg counts [71]. The Florida Native, St. Croix and Targhee breeds showed some resistance to infection with significantly lower fluke burdens compared to the Barbados Blackbelly; however, no breeds showed evidence of acquired immunity to *F. hepatica* [71].

A leading factor in the potential for establishing acquired immunity towards fluke infections is the resistances observed in the Indonesian thin tail (ITT) sheep. ITT sheep can reject *F. gigantica* infiltration 3–4 weeks after the consumption of infective metacercariae and display resistance after a previous exposure; however, they do remain susceptible to *F. hepatica* infections [72,73,74]. Following a 12-week infection, naive ITT sheep had lower implantation rates for *F. gigantica* (12%) than for *F. hepatica* (31%). Then, following an initial 4-week drug-abbreviated *F. gigantica* challenge, the worm recoveries were 0.3%–3% for *F. gigantica* secondary infection and 3.6%–14% for secondary *F. hepatica* challenge [74]. Importantly, acquired resistance is deemed immunological as the administration of an immunosuppressant, dexamethasone, abolishes resistance and results in ITT sheep with prior *F. gigantica* exposure exhibiting fluke burdens (6.4–9% fluke recovery depending on treatment time) comparable to a susceptible sheep breed (9.6% recovery) and substantially higher than non-medicated previously exposed ITT sheep (1.1% recovery) [73].

Traditional studies on the susceptibility of goats to *F. hepatica* and *F. gigantica* infection are scarce. An investigation into pathological and immunohistochemical effects of *F. hepatica* infections in goats observed 9/15 goats dying from infection with trickle doses of a max of 400 metacercariae [21,64]. Extremely high parasitism was detected by Reddington et al. [75] with a 72% parasite recovery per infective dose after a 9-month long infection of naive mixed breed goats [75]. The authors also described no protective response upon secondary *F. hepatica* infection; however, there was no drug treatment between infections, and flukes were categorised by size and allocated as part of the primary or secondary infection, which may have led to incorrect allocations of flukes to the first and second infections [75]. Similarly, Martinez-Moreno et al. [76] determined that fluke take per infection dose was higher in animals after a primary *F. hepatica* infection, as Serrana goats had a 12.6% take after the initial infection of 200 metacercariae and a 15.5% take after infection with 200 metacercariae and a secondary 100 metacercariae infection 6 weeks after the primary infection, and lastly a 10.4% take was observed in the group only receiving the secondary 100 metacercariae infection [76]. They also defined flukes by their size to distinguish fluke numbers from each wave of infection in the re-infected group, noting a significantly lower number of smaller flukes in the group receiving 100 metacercariae, indicating a rapid advancement of flukes in the secondary infection and a potential to miss allocated flukes from the different infections based on size [62]. Furthermore, liver damage is more severe upon re-infection with higher hepatic enzyme levels indicative of liver damage, with both a high *F. hepatica* metacercaria load in one single bolus dose and trickle infections summating to the same load totalling either 200 or 400 metacercariae resulting in similar fluke recovery per infective dose (19.7–24.3%), indicating that protection cannot be stimulated despite a specific IgG and cellular immune response in goats [64].

There is a potential for goats to develop resistance to fluke infections that could be further investigated and exploited to determine resistance mechanisms. Unlike *F. hepatica* infections, goats show an ability to generate resistance to *F. gigantica*, with at least a 50% reduction in parasite burdens after one and two prior drug-negated challenges [63]. Naive Nubian goats showed a susceptibility rate of 39% towards *F. gigantica* after an initial 400 *F. gigantica* metacercariae dose, recovering a mean of 155 ± 12 flukes. However, there were significantly lower fluke burdens following one or two prior drug-abbreviated exposures of 200 *F. gigantica* metacercariae, recovering 96 ± 21 (24% of dose rate) and 73 ± 37 (18% of dose rate) flukes [63]. Moreover, these investigators showed that exposure to irradiated *F. gigantica* metacercariae reduced fluke burdens by 43% in Nubian goats with mean fluke numbers of 88 ± 39 (22% of dose rate) compared to the unimmunised control, 155 ± 12 (39% of dose rate) [77,78]. Unfortunately, this resistance phenomenon has not been further investigated to the levels of ITT sheep to determine the unique mechanisms of resistance in Nubian goats to *F. gigantica* infections.

Available investigations suggest that goats are highly susceptible to *F. hepatica* infections. Goats have a < 25% fluke recovery after *F. hepatica* infection, with no signs of reduced burdens upon secondary infection. However, an initial *F. gigantica* exposure appears to have a priming mechanism in Nubian goats and substantially reduce subsequent infections. This needs to be further explored and exploited for determining resistance mechanisms.

## 5. Vaccinology for Controlling Caprine Fasciolosis

*Fasciola* spp. have an impressive prowess to manipulate their animal host environment for their survival, considering they successfully infect most mammals they encounter and can manipulate multiple host immune systems for their survival. Despite understanding the fundamental immune responses to fluke infection, there is still no well-defined causation of acquired immunity in rodent models or natural hosts [79,80,81,82,83]. The immunological basis of caprine-specific liver fluke killing is poorly understood in comparison to other ruminants [79,84,85].

While individual host species display variations in pathological and immunological responses during fasciolosis, there is a general defined response from fluke infections: an initial mixed type 1 and type 2 helper T cell response (Th1/Th2), followed by Th2 polarisation and a subsequent regulatory T cell response [82,84,86,87,88]. Counteracting this establishment of a Th2 profile, consisting of interleukin (IL)-4, IL-5, IL-10, IL-13 and IgG1 production, and enhancing the Th1 environment, IL-2, interferon (IFN)γ and IgG2, are thought to lead to protection against liver fluke infection [79].

Immunological control of liver fluke could involve swaying the host immune system to a Th1 environment adequate for parasite killing. During infection in Merino sheep, the Th2 polarisation occurs around 9 days post-infection in the hepatic lymph nodes and 18 days post-infection in the liver, with a significant increase in IL-4 expression that suppresses IFNγ levels [89]. Conversely, ITT sheep, which are resistant to *F. gigantica* and susceptible to *F. hepatica*, demonstrate a less dominating IL-4/IFNγ ratio and elevated IgG2 levels when displaying resistance against *F. gigantica* 10 weeks post-infection, compared to during a *F. hepatica* infection [65,74]. These data led to the presumption that a Th1-like immune response is associated with protection against liver flukes. This notion is further supported by the in vitro killing of juvenile *F. hepatica* through antibody-dependent cellular cytotoxicity (ADCC) in a nitric oxide (NO)-mediated manner, which is released by classically activated macrophages in a Th1 cytokine environment [65,90,91]. Therefore, a component to establishing liver fluke immunity is creating a localised Th1-bias, potentially by dampening the effects of Th2-modulating fluke antigens or further stimulating the immune system using a Th1-stimulating adjuvant.

There have been several vaccination trials using goats as the host for protection against *Fasciola* infection. Vaccine antigens tested include cathepsin L proteases (CL1 and CL2), glutathione *S*-transferase (GST) and *Schistosoma mansoni* fatty acid-binding protein (Sm14) (Table 2). Vaccination with a cathepsin L mimotope expressed in a peptide phage display system currently shows the most promise thus far for caprine fasciolosis protection [92,93]. The main adjuvant used has been Quil A, a saponin that induced mixed Th1/Th2 and cytotoxic T lymphocyte responses [94,95].

**Table 2:** Vaccine antigen protection levels in goats.

Parasite Species	Protein	Antigen ^1^	Goat Breed	Delivery Route	Met ^2^ Dose	Group (Number of Goat)	Fluke Implementation Rate %	Reduction in Fluke Burden % (*p*-Value)	Ref.
*F. hepatica*	Cathepsin	rCL1	Florida Servilla	Subcutaneous	200	rCL1 + Quil A (n = 7)	28% ^a^	39%	[96,97]
		rCL1 (pro)	Malaguena	Subcutaneous	100	rCL1 + Quil A (n = 10)	55.9% ^b^	0%	[98]
		Phage CatL1	Goats	Subcutaneous	200	Clone 11 (n = 6)	29.75% ^c^	31%	[92]
						Clone 13 (n = 6)	22.9% ^c^	47% (*p* < 0.05)	
						Clone 13 + Quil A (n = 6)	8.8% ^c^	79.5% (*p* < 0.05)	
		Phage CatL1/L2	Cross breed	Subcutaneous	200	CL1 Clone 7 + Quil A (n = 5)	-	55% (*p* < 0.05)	[93]
						CL1 Clone 13 + Quil A (n = 5)	-	70% (*p* < 0.05)	
						CL2 Clone 10 + Quil A (n = 5)	-	32%	
	Glutathione *S*-transferase	nGST	Florida Servilla	Subcutaneous	200	nGST + FCA/FIA ^3^ (n= 6)	24% ^d^	9%	[99]
		rGSTsigma	Malaguena	Subcutaneous	100	rGSTsigma + Quil A (n = 6)	59% ^b^	0%	[100]
	Peroxiredoxin	rPrx	Florida Servilla	Subcutaneous	200	rPrx + Quil A (n = 7)	31% ^a^	33%	[96,101]
	*Schisotosoma mansoni* fatty acid-binding protein	rSm14	Florida Servilla	Subcutaneous	200	rSm14 + Quil A (n = 5)	51% ^a^	0%	[96,102]
		pSm14	Florida Servilla	Subcutaneous	200	pSm14 + RIBI+ Alum (n = 6)	14% ^d^	46%	[103]
	Combination	rCL1, rPrx, rSm14	Florida Servilla	Subcutaneous	200	rCL1, rPrx, rSm14 (n = 6)	42% ^a^	10%	[96]
*F. gigantica*	Crude extract	Crude	Goats	Immunised	125	Crude + FCA/FIA ^3^ (n = 3)	14.4% ^e^	23.7%	[104]
	Excretory/secretory	E/S	Goats	Immunised	125	E/S + FCA/FIA ^3^ (n = 3)	12.8% ^e^	32.2%	
	Glutathione *S*-transferase	nFgGST	Goats	Immunised	125	nFgGST + FCA/FIA ^3^ (n = 3)	6.4% ^e^	66.1% (*p* < 0.05)	

^1^ n: native protein, r: recombinant, *p*: peptide, ^2^ Met: metacercaria ^3^ FCA/FIA: Freund’s adjuvant system, complete then incomplete. a, b, c, d, e = denotes groups belonging to the same trial. Unless *p* < 0.05 is stated then efficacy is insignificant.

Cathepsin L protease is a major secreted protein from adult *F. hepatica* that has shown great potential as a vaccine candidate and is capable of gaining significant efficacies in cattle (48–69%) but lower efficacy in sheep (34%) [87,105,106]. An essential role of cathepsins L-like proteases during infection is the ability to cleave host immunoglobins, which in turn hypothetically reduces effector cells binding to fluke bound antibodies which would subsequently suppress fluke killing by ADCC [107,108,109,110]. Initial efforts to determine the potential of recombinant cathepsin L (rCL1) as a vaccine in goats with Quil A showed a 39% reduction in worm burden and noted a considerable individual variation in responses; subsequently, it was tested in a more significant number of animals and gained no efficacy [96,97,98]. Despite a lack of efficacy in the latter study, it could still be worth pursuing rCL1 in different adjuvants as immunised animals showed an increased weight gain over infected animals, which has substantial benefits for the production value of the animal [98]. Furthermore, rCL1 vaccination resulted in lower hepatic lesions correlated to increased levels of peritoneal cells expressing induced nitric oxide synthase, which in turn increases NO levels: higher NO levels could have contributed to the vaccination reducing fluke burdens during the peritoneal stage of migration [98].

The highest protection observed in goats against *F. hepatica* is using phage displaying peptides of cathepsin L, gaining efficacies of 31–79.5% and resulting in only an 8.8% implantation rate at its best [92,93]. Phage clones that react with anti-cathepsin L antibodies have been analyzed using sera from vaccinated animals, and vaccination with the 7-mer clone 13 (SGTFLFS) alone and combined with Quil A has induced a statistically significant 47% and 79.5% efficacy, respectively [92]. This vaccination stimulated a mixed Th1/Th2 response reflected by the induction of IgG2 and IgG1 antibodies, where IgG2 levels may be indicative of protection, albeit not directly [79,92]. Furthermore, the phage clone 13 alone and delivered with Quil A stimulated an IgG2 and total IgG (not IgG1) response that significantly correlated to reduced fluke burdens [92]. The efficacy of the peptide of phage clone 13 in Quil A appears reproducible, inducing 70% efficacy and a 74% reduction in egg output in a subsequent goat vaccination trial [93]. This phage display vaccine method is also transferable between host species, gaining 33.9–51% efficacies in sheep [111,112]. The cross-trial reproducibility of vaccine efficacy, transferability between host species and correlation between efficacy and IgG2 levels in goats mean this vaccine formulation is worth further exploration.

The proposed *F. hepatica* cross-reactive anti-helminth molecule from *S. mansoni*, rSm14, has produced high protection in lambs against fasciolosis, almost eliminating flukes from a small sample size of sheep (n = 3–4) [113,114]. Unfortunately, this efficacy was not transferable to goats as the whole molecule vaccination using rSm14 with Quil A could not elicit any protection against fasciolosis [96,101]. However, a 10 amino acid synthetic peptide of Sm14 (EKNSESKLTQ) in the adjuvants (RIBI and Alum) used in the aforementioned rSm14 vaccination sheep study was able to induce 46% efficacy (not significant) in goats, along with fewer T lymphocytes infiltrating into the hepatic lesions relative to the infected control group [103]. In two of the six animals that had zero and five flukes, there was evidence of a low cellular influx to the liver and minimal hepatic lesions, suggesting that flukes were killed early on in infection [103]. IL-4 and IFNγ levels were also depicted in this study, showing low hepatic lymph node IFNγ levels in both the infected and immunised goats, suggesting the Th2 polarisation had occurred in the later stage of infection that is also observed in other goat vaccination trials and in cattle and sheep infections [100,101,102,103,115,116,117,118]. Theoretically, there is potential for the Sm14 peptide to induce significant efficacy in an adjuvant that could generate a more dominating Th1 response, considering it shows a potential reduction in parasite burden within a Th2 environment.

The detoxification enzyme, native glutathione *S*-transferase (nGST), can generate a range of protection in cattle using different adjuvants (0 to 69%) but an average of 43% efficacy using the optimal adjuvant Quil A/Squalene/Montanide 80 [119]. The partial protection observed in cattle for nGST was not transferable to goats, only generating 9% protection but significantly smaller worm size when using the strong Th1 stimulating Freund′s complete adjuvant system [99]. Similar to the lack of protection gained from nGST, which predominantly consists of mu class enzymes, the recombinant sigma class GST showed no protection in goats with Quil A as the adjuvant [100]. This could lead to the conclusion that GST is not a promising candidate in goats; however, it could be premature to completely dismiss this antigen as it has not been awarded the same level of scrutiny as the Morrison et al. [119] study to find an optimal adjuvant for protection in cattle.

Lastly, recombinant peroxiredoxin (rPrx) has been tested in goats against *F. hepatica*, generating a non-significant 33% efficacy as a single antigen and 10% when co-administered with rCL1 and rSm14 [96,101]. The peroxiredoxin molecule plays a role in parasite survival and defence and is capable of recruiting alternatively activated macrophages, which are macrophages that do not produce NO [120]. rPrx with Quil A stimulated a high specific IgG response, and lower expression of IL-4 in the hepatic lesions in the vaccinated goats over infected controls [96,101]. The rPrx+rCL1+rSm14 combination vaccine did not summate the efficacies observed for rCL1 (39%) and rPrx (33%) alone, perhaps from rSm14 alone not inducing protection in that same trial, potentially indicating combination vaccines are not synergetic for goats, but further investigation is required [96].

Studies testing vaccines in goats against *F. gigantica* are limited, with only a single study testing the efficacy of a crude worm extract, excretory–secretory (E/S) products and native FgGST in a limited study using three goats per group [104]. This study showed that a reduction in *F. gigantica* burdens is possible in goats. However, it only showed significant efficacy of 66.1% using nFgGST, which gained higher protection than the non-significant results using crude (23.7%) and E/S (32.2%) fractions emulsified with Freund′s adjuvant system [104]. Vaccination with nFgGST in goats does warrant further investigation, as it was capable of a significant reduction in parasite numbers, gaining burdens of 4, 8 and 12 compared to the average 23.7 flukes in the control group, along with a 90.7% reduction in egg output and stunted physical development in the parasites that were recovered [104].

There have also been in vitro investigations on the function of peripheral blood mononuclear cells (PBMC) after stimulation from *F. gigantica* thioredoxin (rFgTPx), 14–3-3e (rFg14–3-3e), ras-related rab10 protein (rFgRab10) and cathepsin B (rFgCatB) [121,122,123,124]. The proteins influenced various functions of PBMC, all capable of binding to PBMC cells, promoting apoptosis, increasing the production of NO and increasing the production of both Th1 and Th2 cytokines, with the exception of rFg14–3-3e. rFg14–3-3e may play a role in modulating the Th1/Th2 balance as it increased IL-10 and transforming growth factor-beta expression and lowered IL-4 and IFNγ levels [121]. Uniquely, rFgRab10 inhibited PBMC proliferation and enhanced migration along with promoting monocyte phagocytosis, while both rFgTPx and rFg14–3-3e suppressed proliferation, migration and phagocytosis [121,122,123]. Furthermore, rFgCatB uniquely reduced PBMC cell viability and the authors postulate that knowledge on the function of these proteins could be used to develop a synergistic cocktail vaccine against *F. gigantica* infection [124].

### Future Considerations for Caprine Vaccine Development Against Liver Flukes

Rational selection of liver fluke vaccine candidates to test in a host species is heavily influenced by the fact that specific parasite proteins play vital roles in immunomodulation or defence against the host. Therefore, it is likely that cattle and sheep studies will pioneer the new antigen selection for vaccine development, given their economic importance, while goat investigations can advance in finding an optimal adjuvant or delivery method using currently tested antigens.

Top priorities for a goat vaccine towards *Fasciola* spp.:

1. Determine if phage peptide display of *F. hepatica* cathepsins is reproducible enough for commercialisation or for a first-generation vaccine.
2. Reproduce the significant efficacy generated with *F. gigantica* native GST to determine vaccine potential.
3. Expand the adjuvant repertoire used in caprine vaccinology, potentially using the *S. mansoni* fatty acid-binding protein (Sm14).
4. Investigate if mucosal vaccination platforms could improve the protection of goats from fasciolosis.
5. Test new antigens that show promise in cattle and sheep.

Interesting vaccine delivery platforms to investigate in goats are mucosal-based and edible vaccines. Considering a commercial product targeting caprine fasciolosis would be most beneficial to developing countries with less specialised veterinary resources for mass vaccine delivery, there could be a development focus on creating a stable and edible vaccine for easy administration and roll out. Generating mucosal immunity in livestock against liver flukes by vaccine administration at the mucosal surface could be advantageous over traditional parenteral vaccination. Stimulating mucosal immunity activates the immune system at the site that NEJs are exposed to during the early stages of infection and uniquely triggers the production of IgA [125].

A potent mucosal adjuvant that can stimulate a mixed Th1/Th2 response is the AB_5_ *Escherichia coli* heat-labile enterotoxin (LT) and its non-toxic derivatives, such as the B subunit termed LTB [126,127]. The potential use of LTB as a fasciolosis vaccine adjuvant is based on its capabilities of inducing both systemic and mucosal immunity along with a Th1-like immune response (elevated IFNγ and IgG2 levels in mice) when administered via the mucosal route [128,129,130]. LTB interacts with cellular ganglioside receptors, predominantly ganglioside GM1, which can trigger its adjuvant properties [131,132]. The fusion of antigens to LTB can enhance antigen presentation and influence T cell differentiation and immunoglobulin class switching [131,133]. There are minimal data on the potential use of LTB in the ruminant veterinary landscape, especially in terms of the Th1/Th2 balance. However, LTB delivery is known to elevate specific IgG and IgA production in pigs towards *Mycoplasma hyopneumoniae* fused antigens [134]. After intranasal administration in cattle of another fused AB_5_ toxin, the cholera toxin’s B subunit, specific IgG and IgA antibodies were generated and enhanced resistance was observed against *Mannheimia haemolytica* [135]. The subcutaneous vaccination of cattle with LTB linked to a fragment of the botulinum neurotoxin created a greater titre of neutralising antibodies against serotype D than the current commercial vaccine for bovine botulism [136]. Furthermore, plant encapsulated LTB given to sheep following transgenic plant expression could withstand the ruminant digestive system and induce a mucosal humoral response in the abomasum, small intestine and mesenteric lymph nodes [137].

Another edible vaccine system that has been tested against fasciolosis in goats is a hepatitis B core protein bound to a cysteine proteinase cathepsin protein within transgenic lettuce, which generated reasonable efficacy in sheep (35.5%, not significant) and significant efficacy in cattle (56.2%) [138]. Virus-like particles like the hepatitis B core protein can present multiple copies of a linked antigen on its surface, which can crosslink receptors on B cells and stimulate a robust humoral response [139,140]. Another possibility of a mucosal adjuvant for goats is the immune-stimulating complex (ISCOM), which is a cage-like structure consisting of Quil A, cholesterol and phospholipids that can bind and carry antigens [141]. ISCOMs can produce a balanced helper T cell response and a humoral immune response, including mucosal IgA [142]. Sheep intranasally immunised with ISCOM and CpG containing CatB2 and CatL5 had a significant reduction in faecal egg output (95%) and higher protective levels than intramuscular delivery of the same antigens in just Quil A, gaining a significant 45.5% compared to the non-significant 20.9%, respectively [143]. Given the success of some antigens in Quil A for goats, ISCOM would be interesting to analyse as a vaccine adjuvant against fasciolosis.

Generating effective long-term immunity against *F. hepatica* and *F. gigantica* still eludes scientists, even in the more investigated species such as cattle and sheep. Considering liver flukes have an impressive ability to infect and modulate a large range of mammals and their immune systems, an all-encompassing cross-species fasciolosis vaccine might not be feasible. Therefore, caprine vaccinology should not continue to be overlooked since protection across species is not simple transferable. Controlling goat infections in highly prevalent or vulnerable areas could consist of a first-generation cathepsin phage display vaccine since it shows considerable promise thus far. It seems apparent that when vaccine protection occurs, it targets immature flukes before arriving at the liver since efficacy is observed alongside reduced liver damage. Therefore, aiming to stimulate mucosal immunity at the site where the infection is established and the immature flukes reside could improve current vaccine efficacies. This could comprise an edible vaccine formulation, which could also be a practical approach for rolling out a vaccine in developing countries. There are also extensive benefits in advancing the potential for an anti-helminth vaccine in goats using the peptide of *S. mansoni* fatty acid-binding acid, which could have potential in a commercial product if efficacy is proven reproducible with an optimal adjuvant pairing. Combining the potential requirement for a mucosal vaccine and a Th1 driving adjuvant highlights the potential for the heat-labile enterotoxin B subunit from *E. coli* to stimulate the desired responses in both facets. Finally, immunomodulatory molecules from *F. gigantica* are in dire need to be tested in goats since there are scarce investigations in goats vaccinated with an *F. gigantica* molecule.

## 6. Current Standings on Controlling Liver Fluke Infections in Goats 

Vaccine development for the control of fasciolosis in goats is ongoing and will be a future applicable control method, whereas anthelmintics are the current critical frontline control option. Drug treatments in goats should be tightly monitored and correctly administered to prolong drug efficacy and avoid the continual emergence of drug-resistant liver fluke populations [2]. The determination of efficacy for some flukicides has been completed in goats, which uniquely metabolise and eliminate drugs quickly from their bodies compared to sheep and cattle [76,144,145,146,147,148,149]. Strategic farming management plans could be essential to continually control caprine fasciolosis and avoid goats contributing to generating resistance to anthelmintics. However, in regions with scarce veterinary assistance that use basic farming practices, natural anti-parasitic plant extracts and fungal species could be exploited for fluke control in goats.

There are a number of drug classes that can treat liver fluke infections, with readily available information on efficacies towards different parasite life stages for cattle and sheep [150]. Efficacies of treatment in goats have been assessed via controlled studies, challenges, and post-mortems for triclabendazole, albendazole and clorsulon [76,144,145,146]. However, efficacies for nitroxinil, closantel and oxyclozanide have only been determined using faecal egg counts, which do not directly correlate to parasite numbers [151,152,153]. Therefore, treatments with these latter three drugs should be further investigated before application for caprine fasciolosis control. Recent reviews focusing on cattle and sheep have expertly covered the global occurrence of drug-resistant fluke populations, integrated pasture management strategies, drug-resistant liver fluke isolates, and potential mechanisms of drug resistance, along with stating the efficacy of commercial therapeutics towards different life stages of flukes [1,2,81,150,154]. Herein, we will cover similar topics but with a caprine focus.

Triclabendazole (TCBZ) is the most effective drug for fluke control on the market, capable of killing both immature and mature life stages of the liver fluke [150]. This is also the case in goats, where treatment of Sarrana goats at 10 mg/kg of body weight after a 200 *F. hepatica* metacercarial challenge provided efficacies of 94.9% and 99–100% after 4- and 8-weeks post-infection, respectively [76]. TCBZ killing at 10 mg/kg also occurs for immature *F. gigantica* in caprine infections, where treatment was highly effective three days after oral treatment [146]. Due to its superiority in killing multiple fluke life stages, TCBZ has been heavily used and now has the widest spread of resistant fluke populations [1,2]. There has been a case of TCBZ resistance in fluke-infected goats in Brazil where TCBZ treatment failed to efficiently reduce egg output [155]. There is also a report of TCBZ resistance in cattle in Argentina during 2008 [156]. Goats in Argentina showed a 33% fluke infection prevalence during 2006–2011; this could indicate future drug resistance populations [31]. However, TCBZ treatment still appears to be effective in crucial caprine fasciolosis locations, with recent successful treatments in sheep, cattle or buffalos in Pakistan [157,158], China [60], Iran [159], Egypt [160] and Ethiopia [161].

Both albendazole and clorsulon show therapeutic effects in goats against adult flukes through post-mortem examination after drug treatment [144,145]. In both studies, goats were treated after 14 weeks of an established *F. hepatica* infection and found treatment efficacies of 98–100% for clorsulon at dose rates of 7–15 mg/kg and 88–96% for albendazole at doses of 7.5–15 mg/kg [144,145]. Comparatively, fluke-infected goats show similar efficacies for albendazole and clorsulon to cattle and sheep harbouring flukes older than 14 weeks. An investigation is required to determine if the killing of flukes < 14 weeks old in goats occurs from albendazole and clorsulon treatment. While the potential for effective drug treatment of fluke infection is likely translatable between hosts, adequate dosage rates need to be set for each host species. Goats have an increased metabolic rate and can eliminate drugs quickly through their system, leading to a lower bioavailability [162,163]. Clorsulon has a faster elimination rate in goats compared to sheep after oral administration, where urine excretion contained 40% of the clorsulon dose rate for sheep in approximately 20 h, whereas goats eliminated 45% of the dose rate in 10 h [147]. Furthermore, goats have a significantly shorter half-life of absorption for closantel, being 4 days compared to 14 days as is seen in sheep [148]. This ultimately suggests that the goat′s increased rate of drug metabolism results in less exposure time between the drug and the parasite, and therefore less time for the drug to cause detrimental effects upon the parasite.

Albendazole or closantel resistance in liver flukes residing in goats has not been reported in the literature. However, there has been a case of efficacy loss in Egypt using albendazole and rafoxanide in fluke populations isolated from cattle [160]. This fluke drug-resistant population could be easily transmitted to locally farmed goats. Goats also quickly metabolise albendazole sulphoxide (ABZ-SO), with a significantly lower plasma half-life and bioavailability compared to sheep [149]. Therefore, to counteract the lower exposure time between the parasite and drug, the authors investigated increasing dosage rates, which did increase the total plasma levels of albendazole sulphoxide in goats [149]. However, the effectiveness of increased dose rates in goats needs to be monitored and assessed as underdosing fosters resistance [163]. Even accounting for increased dose rates, parasite drug resistance was promoted towards monepantel, with goats labelled as a significant contributor to resistance [164]. On a New Zealand farm, monepantel was used to treat roundworms in goats at 1.5×, and then subsequently 3×, the sheep dosage rate but failed to provide efficacy, only two years after first being used [164]. Both monepantel and its sulphone form can be active anthelmintics, with the latter having a shorter half-life in goats compared to sheep, reducing its exposure period to the parasite, reducing efficacy, and this could possibly have led to the induction of drug-resistant roundworm populations [164]. The generation of drug-resistant parasites by underdosing goats affects other co-grazing livestock species, and this could have detrimental effects for the control of fasciolosis.

The seasonal prevalence of goat fasciolosis needs to be determined in the location where control is required to account for seasonal trends and treatment timing. Higher prevalence of liver flukes is frequently noted in winter or the wet season for goats, generally when sheep also showed high prevalence [35,36,43,48,55]. Therefore, treatment timing for fasciolosis can be inferred from sheep data, with an initial treatment of TCBZ (if available) during peak infection season and then subsequently a secondary treatment targeting adult flukes and egg production towards the end or just after peak ingestion season [2,163]. Rotating drug classes with different modes of action or using drugs in combination can help suppress the development of resistance; however, efficacies and synergistic effects of drug combinations have not been investigated in goats. The choice of drug used likely depends on what drug products are available in that region and what regulations are set in place. Effective combinations and new promising drugs for treating fasciolosis in other ruminant hosts have been summarised recently in Fairweather et al. [2], which presented new drug formulations that warrant investigation in goats to improve the solubility and, subsequently, the bioavailability of currently used drugs. The benzimidazoles have low aqueous solubility, but solubility has been improved with compounds such as alpha triclabendazole and MFR-5, a triclabendazole prodrug, but these two drugs have only been tested in cattle or sheep [165,166,167]. The evaluation of improved formulations should be considered a priority to see if success can be replicated in goats.

### 6.1. Ecological Control

The microbiota of helminths could be an avenue to exploit for parasitic control. Parasite endosymbionts can utilise the parasite as a vector to cause pathogenesis within mammalian definitive hosts, which could be the case for *Neorickettsia* identified within the *F. hepatica* Oregon isolate [168]. Genomic analysis aligns the fluke inhabiting *Neorickettsia* to species which cause severe illnesses in dogs, horses and humans [168]. Although not established, it infers a potential for fluke microbiota to exacerbate health issues within fluke-infected ruminants. Conversely, further investigation into the liver fluke microbiome could identify novel parasite control strategies. The disruption of key symbiotic bacteria could reduce parasite propagation. Antibiotic therapy targeting *Wolbachia*, which filarial worms rely on for reproduction and survival, decreases the associated inflammation and river blindness in humans by rendering the parasites sterile and reducing the continuation of the parasite life cycle [169,170,171].

There are practical farming practices that can be immediately implemented to prolong the activity of commercial drugs and control fluke infections in goats. Recently, the development of molecular tests for identifying environmental DNA (eDNA) from *F. hepatica* or intermediate host snails (*A. tomentosa* and *G. truncatula*) can allow the accurate determination of fluke-contaminated areas on farms [172,173,174]. Monitoring eDNA shed from free-living life stages of flukes or transmitting snails would allow for precise identification of transmitting zones and snail habitats for sound advice on the use of molluscicides and fencing off regions on farms. The site-specific small-scale use of molluscicides after eDNA detection of a substantial abundance of fluke transmitting snails could reduce the environmental impacts that are associated with large-scale molluscicide application [175]. Educating farmers in Switzerland about fasciolosis so they could apply grazing strategies, along with reducing livestock exposure to contaminated pasture and snail habitats, was shown to have substantial benefits in decreasing infections of gastrointestinal nematodes in cattle [176,177]. Farmers that had the motivation and capabilities to take on the recommendations saw a decrease in positive faecal samples from 30.7% to 9.3% over the 4-year period. Conversely, farms that partially, incorrectly or were entirely unable to implement management styles had no reduction in egg counts [177]. A high proportion of goats in developing rural areas are loosely managed and have inconsistent veterinary services; therefore, this control scenario might not be feasible or widely adopted in caprine farming in developing countries.

### 6.2. Biological Control Using Predatory Fungus

A form of biological control is using fungi species that can quench the free-living stages of parasite larvae and thus decrease the infective parasitic stage and transmission to ruminants. These predatory fungi could be supplemented into feed and allowed to pass through the gastrointestinal tract of ruminants and into the faeces to destroy helminth eggs [178]. In vitro analysis of *Verticillium chlamydosporium* and *Pochonia chlamydosporia* revealed both could be grown in a laboratory setting and were capable of destroying viable eggs of *F. gigantica* and *F. hepatica*, respectively [179,180,181]. A field trial in Brazil applied *P. chlamydosporia* into cattle feed (1 g/10 kg bodyweight) twice a week for 18 months. This treatment showed reduced fluke egg counts in faecal matter in the latter six months of the experiment because the fungus successfully passed through the ruminant gastrointestinal tract and could retain activity towards *F. hepatica* eggs [180,182]. The fungus *Duddingtonia flagrans* can reduce nematode infections in cattle, sheep and goats with sufficient efficacies translated to a commercial product BioWorma^®^. Fungal products for biological control could be used to control fasciolosis due to their ease of application and affordability, making this a feasible control method in developing countries.

### 6.3. Natural Plant Extracts

Natural plant-based extracts have been used in places such as Africa for their anthelminthic properties as an alternative to drugs. Plants contain phytochemicals such as alkaloids, flavonoids, phenols, saponins and condensed tannins that show activity against helminths [183]. Indigenous plants with such properties have been outlined in Mazhangara et al. [183], denoting known efficacies and modes of action. Control of *H. contortus* and *F. hepatica* infections in Pakistan was achieved using a herbal deworming mixture in goats that could significantly reduce both *F. hepatica* egg (82.35%) and *H. contortus* egg (91.35%) counts compared to untreated animals [184]. The plant material was locally purchased and ground and mixed, containing Chinese honeysuckle, fennel, linseed, and Viper’s bowstring hemp [184]. Elephant root or *Elephantorrhiza elephantina* has also been used to control *H. contortus* infections in goats, reducing egg counts comparable to the commercial drug used in South Africa [185]. The use of herbal extracts to control fasciolosis in goats could be an invaluable resource that requires evaluation.

## 7. Conclusions

Controlling parasite infections in livestock is crucial for the future sustainability of agriculture. Infections caused by liver flukes have substantial impacts on goat production systems, which has been overlooked compared to cattle and sheep. Further investigations into the potential immunity mechanisms evident in Nubian goats towards *F. gigantica* could be invaluable for future control. Caprine fasciolosis′s global prevalence and economic impacts can only be surmised as levels are likely not genuinely equated in the literature. It is essential to monitor caprine liver fluke infections and the effectiveness of control methods. Goats have a lower bioavailability of therapeutic drugs compared to sheep; therefore, avoiding underdosing is critical in goats so fluke drug resistance is not generated. It is unlikely that an all-encompassing cross-species fasciolosis vaccine will be established. Therefore, the development of a vaccine also needs to include caprine efficacy studies. While research is limited, there is promise in a phage peptide display method of *F. hepatica* cathepsin that has induced significant protection. This formulation warrants further investigation. It is also worth exploring mucosal vaccine platforms that could aid in generating resistance at the site where the infection is established. Alongside vaccinology, research into potential biological control methods, such as predacious fungal species, could be implemented in developing countries that have high numbers of goats and limited veterinary services.
